# Health literacy, pain intensity and pain perception in patients with chronic pain

**DOI:** 10.1007/s00508-017-1309-5

**Published:** 2018-01-10

**Authors:** Philipp Johannes Köppen, Thomas Ernst Dorner, Katharina Viktoria Stein, Judit Simon, Richard Crevenna

**Affiliations:** 1 Centre for Public Health, Department of Social and Preventive Medicine, Medical University Vienna, Vienna, Austria; 2 Landesklinikum Hochegg, Grimmenstein, Austria; 3 International Foundation for Integrated Care, Oxford, UK; 4 Centre for Public Health, Department for Health Economics, Medical University Vienna, Vienna, Austria; 5 Department for Physical Medicine, Rehabilitation and Occupational Medicine, Medical University of Vienna, Vienna, Austria

**Keywords:** Educational status, Pain management, Self-management, Patient participation

## Abstract

**Background:**

Chronic pain poses a large burden for the healthcare system and the individuals concerned. The impact of health literacy (HL) on health status and health outcomes is receiving more and more attention. The aim of this study was to evaluate the association of HL with chronic pain intensity and pain perception.

**Methods:**

A total of 121 outpatients suffering from chronic pain (pain duration >3 months) were evaluated. The HL was measured using the health literacy screening questions. Pain intensity was measured with a Visual Analogue Scale (VAS) and pain perception with the short-form McGill Pain Questionnaire (SF-MPQ).

**Results:**

Individuals with low HL had significantly higher VAS values (Pearson correlation coefficient= −0.270, *p* = 0.003). Stepwise regression analysis showed that HL has a significant association with pain intensity (odds ratio [OR] = 2.31; 95% confidence interval [CI] 1.11–4.83), even after controlling for age and sex (OR = 2.27; 95% CI 1.07–4.82), but no longer after controlling for education (OR = 2.10; 95% CI 0.95–4.64).

**Conclusion:**

Individuals with a higher HL showed less pain intensity, which seems to be caused by a better pain management; therefore, supporting the development of HL in patients with chronic pain could be seen as an important objective of integrated care.

## Introduction

Chronic pain causes huge financial costs for the society and imposes a significant emotional and physical burden on the individuals concerned [1]. The prevalence of chronic pain in Austria is 21%. The average pain duration is 5.8 years, 31% of the persons surveyed in Austria said that their pain is not adequately controlled and only 16% had seen a pain management specialist. In comparison, the prevalence of chronic pain in Europe is 19% [2]. Chronic pain causes high direct costs through therapies and high indirect costs through reduced productivity of the patients [3, 4]. Out-of-pocket costs for individuals with chronic pain in Austria amount to 2349 € a year and the persons concerned have an extra expenditure of more than 5 h per week for consultations and therapies [5]. The persons concerned also have a reduced quality of life [6].

Health literacy (HL) is defined “as the cognitive and social skills which determine the motivation and ability of individuals to gain access to, understand and use information in ways which promote and maintain good health” [7]. In the European health literacy survey (HLS-EU), the biggest survey about HL in Europe so far, 18.2% of the Austrian population had an inadequate HL, 38.2% a problematic HL, 33.7% a sufficient HL and 9.9% an excellent HL. Of the eight surveyed countries, only Bulgaria and Spain had poorer results. Predictors for low HL are financial deprivation, low social status, low education and old age [8].

Low HL is associated with poorer health-related knowledge and comprehension, increased hospitalizations and emergency care and especially among elderly persons as shown by poorer overall health status and higher mortality [9]. According to Mackey et al. [10], low HL influences the development of self-management skills which are key resources for the treatment of chronic diseases. There is only little literature about the impact of low HL on chronic pain. In a study from Devraj et al. it was found that in a group of patients with chronic pain the persons with low HL had poorer pain medication knowledge, did not know where to find health care professionals to help them manage their pain, and had very little knowledge of non-medication modes of treating pain. No impact of HL on pain intensity was found [11]. In an Australian study from 2010, HL and beliefs about pain between groups with and without chronic low back pain were compared. There was no difference in HL as measured with the Short Test of Functional Health Literacy in Adults (S-TOHFLA) between the groups but in a qualitative analysis of interviews, the group with low back pain reported difficulties in seeking, understanding and using medical information about chronic low back pain [12]. Loke et al. [13] found no consistent association between low HL and poorer functional outcomes in their systematic review about patients with chronic musculoskeletal diseases. The heterogeneity of existing findings suggests a potential influence of cultural and health care system contexts on the relevant association between HL and pain management skills. The current study aimed to assess whether there is an association between HL on pain intensity and pain perception in a heterogeneous group of patients with chronic pain in Austria.

## Methods

Outpatients suffering from chronic pain (with a pain duration more than 3 months) were included, with the goal to evaluate the association of HL with chronic pain intensity and pain perception.

After piloting, data were collected in three outpatient departments (Clinic for Physical Medicine and Rehabilitation, Headache Outpatient Clinic of the Department of Neurology, Pain Outpatient Department of an Orthopedics Hospital) in Vienna between December 2012 and February 2014. The survey took place within the study “Chronic pain and its development depending on social environment, health literacy and previous treatment of the patients and the costs incurred”. In the Clinic for Physical Medicine and Rehabilitation, all patients who fulfilled the inclusion requirements were informed about the survey by the physicians after standard consultation. If they agreed to participate and signed an informed consent, they were interviewed face to face with a predesigned questionnaire by a medical student as part of the same appointment. From December 2012 to May 2013 44 patients were interviewed in the Clinic for Physical Medicine and Rehabilitation. The feasibility of the same approach was questioned during piloting between June and July 2013 in the Headache Outpatient Clinic; therefore, here a different recruitment protocol was implemented. All patients treated from January 2012 till July 2013 in the clinic were informed about the survey in a letter and contacted by telephone afterwards and an appointment for the interview was agreed with the persons interested. A total of 22 patients of the Headache outpatient clinic were interviewed. In the orthopedic hospital, a total of 55 patients were recruited in the outpatient department as well as in the ward between November 2013 and February 2014. The patients who fulfilled the inclusion criteria were informed about the survey by a medical student and interviewed face to face after they signed an informed consent. The inclusion criteria in all centres were:

- Chronic pain (at least 3 months duration)
- Age between 18 and 65 years
- Sufficient knowledge of the German language
- No current malignant disease
- No current inpatient treatment in a psychiatric ward

The HL was measured using three HL screening questions from the publication “Brief Questions to Identify Patients with Inadequate Health Literacy” [14]. The three questions “how often do you have someone help you read hospital materials”, “how confident are you filling out medical forms by yourself” and “how often do you have problems learning about your medical condition because of difficulty understanding written information?” were translated into German using the method of retranslation. The answers on the 5‑point Likert scales were rated with 1 point for the worst answer option and 5 points for the best answer option. A total score was calculated, with 15 points as the best possible value and 3 points as the worst possible value.

To assess the source where patients get their information about pain from, the following question was asked: “from where do you obtain information about your pain disease?” Multiple answer options were possible including: doctor/journal/TV/radio/internet/informative lectures/self-help groups/family and friends/other.

Pain intensity was measured using a Visual Analogue Scale (VAS) [15]. Pain perception was measured using the short-form McGill Pain Questionnaire (SF-MPQ). Besides pain intensity, the SF-MPQ also records sensory and affective components of pain. The SF-MPQ consists of three components. The main component is made up of 11 sensory and 4 affective descriptors. The descriptors are rated from 0 (none) to  3 (severe), this leads to a maximum score of 45 in this component. The second component is the present pain intensity index (0 to 5 points) and the third the VAS. The total added score ranges from 0 to 60 points [16]. No established critical cut-off points exist for interpretation. A higher score means worse pain [17]. For this study, the German version of Oesch et al. [18] was used. Pain duration was listed in months. Pain localization was elicited with the question “on which parts of the body does the pain occur?” To answer the question, the participants were presented a homunculus with 14 defined parts of the body, with multiple answers possible. General sociodemographic data collected included age, sex, country of birth, highest level of education, monthly household income, employment status and sick leave/in-patient care or rehabilitation in the last 3 months.

A correlation analysis was performed for HL, pain intensity, pain perception and pain duration. In order to quantify the possible influence of the HL on the pain parameters, three stepwise logistic regressions were performed with the dependent variables pain intensity (VAS), pain perception (SF-MPQ) and pain duration. The independent variable was HL and co-variables sex, age and education. Because none of the parameters HL (Kolmogorov-Smirnov 0.000), pain intensity (Kolmogorov-Smirnov 0.001), pain perception (Kolmogorov-Smirnov 0.000) and pain duration (Kolmogorov-Smirnov 0.000) were normally distributed, these four parameters were dichotomized by the median. To investigate the influence of the co-variables sex, age and education at HL a logistic regression with HL as dependent variable was performed. All statistical analysis were performed using SPSS Statistics 19.

The study was approved by the Ethics Committee of the Medical University of Vienna (No. 1624/2012) on 04/03/2013.

## Results

A total of 121 participants completed the questionnaire. For demographic data see Table 1. The HL score had a median of 13, a maximum of 15 and a minimum of 5. Table 2 shows the findings for the three HL screening questions. It turned out that for each of the three questions, over 60% of the participants chose the two best answers. For two of the questions (“how confident are you filling out medical forms by yourself?” and “how often do you have problems learning about your medical condition because of difficulty understanding written information?”) more than 50% chose the best possible answer. The descriptive statistics for the different pain parameters are shown in Table 3.

**Table 1:** Descriptive statistics of demographic data of the study population

Parameter	Value
*N*	121
*Age in years, mean (SD)*	48.8 (9.9)
*Female, N (%)*	89 (74)
*Highest level of education, N (%)*	
Compulsory school	21 (17)
School leaving examination/apprenticeship	74 (61)
University	26 (22)
*Country of birth, N (%)*	
Austria	91 (75)
EUª states other than Austria	17 (14)
Non-EU states	13 (11)
*Monthly household income, N (%)*	
501–1000 €	7 (6)
1001–1500 €	19 (16)
1501–3000 €	47 (39)
>3000 €	42 (34)
No statement	6 (5)
*Employment status (multiple answers), N (%)*	
Employed	70 (58)
Unemployed	21 (17)
Apprenticeship	3 (3)
Homemaker	6 (5)
Retired	24 (20)
*Sick leave/inpatient cure or rehabilitation in the last 3 months, N (%)*	
Yes	57 (47)

ªEuropean Union

**Table 2:** HL screening questions

Question/*answer options*	No. of answers, *N* (%)
*“How confident are you filling out medical forms by yourself?”*	
Extremely	67 (55)
Quite a bit	36 (30)
Somewhat	12 (10)
A little bit	4 (3)
Not at all	2 (2)
*“How often do you have problems learning about your medical condition because of difficulty understanding written information?”*	
Never	61 (51)
Occasionally	25 (21)
Sometimes	19 (16)
Often	13 (11)
Always	2 (2)
*“How often do you have someone help you read hospital materials?”*	
Never	38 (32)
Occasionally	37 (31)
Sometimes	26 (22)
Often	13 (11)
Always	6 (5)

**Table 3:** Descriptive statistics of pain data

Parameter	Value
*Pain intensity, VASª*	
Min.	0
Max	100
Mean	39.8
SD	25.8
Median	40.0
*Pain duration, months (years)*	
Min.	3 (0.25)
Max.	606 (50.5)
Mean	182 (15.2)
SD	172 (14.3)
Median	120 (10)
*Pain perception, SF-MPQ* ^*b*^	
Min.	5
Max.	50
Mean	23
SD	9.5
Median	21
Mean of sensory dimension	11
Mean of affective dimension	4.5
Mean of present pain intensity	3.3
*Pain localization, N (%)*	
Headache and migraine	43 (36)
Face/masticatory muscle/jaw joint/ear	25 (21)
Neck/cervical spine	62 (51)
Shoulders	54 (45)
Upper arms/elbows/forearms	31 (26)
Fingers and hands	28 (23)
Chest	4 (3)
Abdomen/stomach	10 (8)
Back/thoracic spine	30 (25)
Lower back/lumbar spine	70 (58)
Lower abdomen	5 (4)
Hips	39 (32)
Thighs/knees/lower legs	61 (50)
Feet/toes	35 (29)
*“Did a doctor establish a diagnosis for your pain?”, N (%)*	
Yes	85 (70)
*“I do not see a cause for my pain.”, N (%)*	
Yes	25 (21)

ªVisual Analogue Scale

^b^Short-form McGill Pain Questionnaire

The correlation analysis showed that higher values of the HL score correlated borderline moderately with lower values of the VAS (Pearson correlation coefficient = −0.270, *p* = 0.003). Between the HL score and the SF-MPQ no correlation could be found (Pearson correlation coefficient = −0.022, *p* = 0.814) and the HL score and pain duration practically did not correlate with each other either (Pearson correlation coefficient = 0.083, *p* = 0.369).

A correlation analysis was performed for HL score and the co-variables sex, age and education (Table 4). It was found that there was no correlation between the parameters and thus no multicollinearity between the covariables and HL and among themselves.

**Table 4:** Correlation analysis of HL score and covariables

		HL score	Sex	Age	Education
HL score	Spearman-cc	1	−0.052	−0.125	0.151
	*p*		0.571	0.172	0.099
Sex	Spearman-cc	−0.052	1	−0.099	0.012
	*p*	0.571		0.278	0.893
Age	Spearman-cc	−0.125	−0.099	1	−0.055
	*p*	0.172	0.278		0.550
Education	Spearman-cc	0.151	0.012	−0.055	1
	*p*	0.099	0.893	0.550	

*Spearman-cc* Spearman correlation coefficient

The findings of the stepwise logistic regression analysis showed a significant effect of HL on pain intensity. The effect was still significant after controlling for sex, as well as after controlling for sex and age. After additional control for education the OR was 2.10, though the effect was not significant anymore. In contrast to these findings, there was no significant effect of HL on pain perception and on pain duration (Table 5).

**Table 5:** Stepwise logistic regression analysis with independent variables pain intensity (VAS_dich), pain perception (SF-MPQ_dich) and pain duration (pain duration_dich)

	Crude (HLª_dich)	Controlled for sex	Controlled for sex and age	Controlled for sex, age and education
*VAS* ^*b*^				
OR (95%CI)	2.31 (1.11–4.83)	2.28 (1.08–4.81)	2.27 (1.07–4.82)	2.10 (0.95–4.64)
R^2^	0.056	0.090	0.090	0.139
*SF-MPQ* ^*c*^				
OR (95%CI)	1.18 (0.57–2.44)	1.21 (0.58–2.51)	1.10 (0.52–2.33)	1.11 (0.51–2.43)
R^2^	0.002	0.017	0.061	0.062
*Pain duration*				
OR (95%CI)	1.38 (0.67–2.84)	1.36 (0.66–2.81)	1.53 (0.72–3.24)	1.69 (0.77–3.72)
R^2^	0.008	0.012	0.073	0.094

ªhealth literacy

^b^Visual Analogue Scale

^c^short-form McGill Pain Questionnaire

**Fig. 1:**
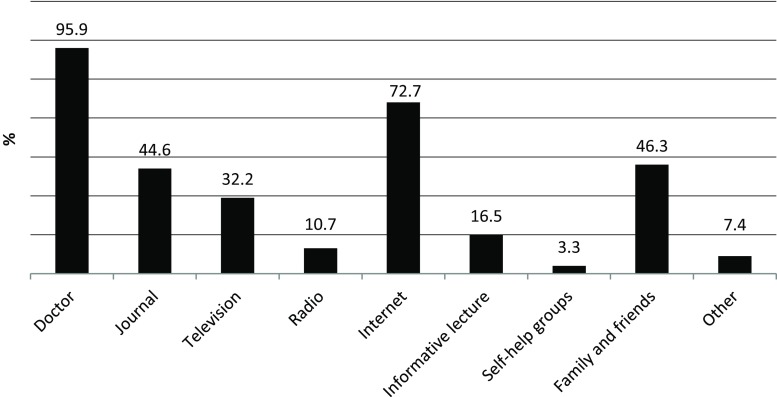
Prevalence of the usage of sources to obtain pain-related information with multiple answers possible

Fig. 1 shows the sources of information as used by the participants. For patients with chronic pain, the general practitioner was the most frequently mentioned source followed by the internet. Self-help groups were mentioned least.

## Discussion

Lower HL was associated with higher rates of pain intensity as measured on the VAS. There was no correlation between HL and pain perception. No association was found between HL and pain duration either. Although the relationship between HL and pain intensity is not clear so far, there are indications that pain intensity is influenced by low knowledge about pain management and poor coping strategies of persons with lower HL [11, 19]. Also, Van Hecke et al. [20] found an association between higher pain intensity and inadequate HL. It is also known that knowledge translation (KT) can help to reduce the burden of persistent pain [21]. Knowledge is a key component of behavioral change and the success of chronic pain treatment is affected by the motivation and ability of the patients to implement these changes in daily life. The KT interventions can lead to positive effects on patient function in short-term and medium-term; however, it is not clear whether other outcomes are also improved by KT interventions [22]. When one considers that patients report difficulties to understand their physicians because of the complex medical jargon used [12], it seems important to implement strategies to enhance patient comprehension. These strategies consist of jargon-free communication, the usage of pictorial aids, the targeted communication of key points at every consultation, and the usage of the “teach-back” method to confirm patients’ understanding [23]. Another aspect that should receive attention is the fact that it is most challenging to get access to qualitative information for individuals with the lowest levels of HL. Furthermore, for physicians it is most challenging and time-consuming to enhance comprehension and HL of persons with low basic skills in literacy and numeracy [24].

Our findings on the large difference between the influence of HL on pain intensity and pain perception leads to the question of the measurement validity of these two parameters and may help to choose which one is more important to measure in patients with chronic pain. The main difference between the VAS and the SF-MPQ is that the SF-MPQ measures, besides the quantity of pain intensity, additionally the quality (sensory and affective) of pain [17]. Zalon [25] suggested that these instruments measure different aspects of pain. The results can be influenced by the instructions given to the patients. A study with patients after radical prostatectomy showed that the group of older men had lower scores on the MPQ than the younger group while there were no differences on the VAS [26]. According to Coll et al. [27] the SF-MPQ is more appropriate to measure pain intensity than pain duration and pattern over time. Since all these findings result from research about postoperative pain and not about chronic pain it stays unclear why there were such big differences between the VAS and the SF-MPQ, taking into account that the VAS is integral part of the SF-MPQ. But there is evidence that pain intensity can differ significantly between different pain scales [28].

Limitations in the measurement of pain in chronic pain patients are a confusion about the definition of pain, a difficulty of averaging pain and the fact that pain measurement is influenced by things other than pain. The confusion about the definition is relevant especially for patients with neuropathic pain who are uncertain whether paresthesia and uncomfortable numbness should also be referred to as pain. Secondly, the difficulty of averaging pain is important for chronic pain, because the pain level often varies considerably between different days and also during a single day. Thus, it is difficult to appoint the intensity of the pain with a single value. Thirdly, patients often incorporate dimensions of pain (e. g., affect, interference) and constructs outside of the pain experience (e. g., what the interviewer might think) into their pain intensity ranking [29].

In our findings the level of education had a significant influence on the level of HL. After control for education, the influence of HL on pain intensity was not significant anymore<, therefore, the level of education affects the pain intensity of the patients. These findings are in line with the findings of other authors [30, 31]. Well-educated persons have a better self-management of pain therapy and better coping-strategies. Van der Heide et al. [32] found that HL has the role of a mediator in the relationship between education and health.

There was no correlation between HL and pain duration. The fact that HL has an effect on pain intensity but not on pain duration can be explained through the continuous development of HL in people with chronic conditions. The development of HL is strongly influenced by the healthcare professionals the patients consult with their pain. Patients can be encouraged by their doctors to engage with information about their disease and actively take part in decisions about their therapies. Other factors that help increase HL are a high personal motivation and fear of negative consequences of the disease or operations. Factors that inhibit the development of HL are lack of personal motivation, non-acceptance of the diagnosis and passing the responsibility onto the physicians [33].

To assess the relationship between HL and chronic pain it is also important to look at the sources from where the patients got their information. The frequency distribution for the question “from where do you obtain information about your pain?” showed that the majority of patients have confidence in their doctors when it comes to where to get information about their disease: but it is important how much of the given information is understood and remembered by the patients. Many patients have problems to understand the instructions from their physicians [12]. Most common mistakes made by physicians are the use of medical jargon, delivering too much information at one encounter, and to miss confirming patients’ understanding. In medical educations for students as well as for practitioners, HL and strategies of clear patient communication should be implemented [34]. In their Health Literacy Conference Report 2008 the European Patients Forum (EPF) [35] recommended development of a guideline to help health care professionals providing user-friendly information for the average citizen. Particularly suitable to enhance patients’ understanding is the combination of visual with written or oral information [35].

The internet has an increased importance as a source of health information in our study in comparison to earlier studies [36, 37]. This finding could be expected, because the use of the internet in the population of industrial countries increased rapidly in the last years, in Germany from 37.0% in 2001 to 76.8% in 2014, for example [38]. Individuals with a high education status use the internet more often as a source than those with a low education status [36]. Less than half of chronic pain patients who use the internet for pain-related information discuss their own findings with their physicians [37]. This may be because the quality of the information found on the internet is often unclear and patients may not understand it enough to actively use the information. Only 3.3% of the participants received their pain-related information from self-help groups. This may indicate that chronic pain patients hardly use self-help groups and there is only little knowledge transfer between individuals concerned with chronic pain. According to Dierks et al. [39], self-help groups play an important role for individuals with chronic conditions. The progressive establishment of self-help groups can be seen as a reaction to a deficiency to provide information by the healthcare system. Evidence about the utilization of self-help groups generally and in particular for chronic pain patients is rare [40]. This field needs to be investigated profoundly considering that group medical visits can help patients to reduce pain through pain education, improved self-management, and the exchange with other chronic pain patients [41].

### Limitations

Limiting factors on validity of the study are the inclusion criteria “Age between 18 and 65 years” and “sufficient knowledge of the German language”. Thus, two high risk groups for low HL, older people and persons with poor ability to speak and read German, were excluded from the study. The measured HL in the study population might be higher than in a population without these exclusion criteria. This distortion could be reinforced by the self-selection bias of the recruitment. The health literacy screening questions were translated into German using the method of retranslation; however, it is not a validated instrument to measure HL in a German speaking population. Another bias could result from the fact that the interviews were performed by different persons in different places under different circumstances. Furthermore, self-reported measures of HL could be biased by the influence of shame of the participants [42].

## Conclusion

Our findings confirm the mediator effect of HL for pain intensity but not for pain perception and pain duration. A higher HL could empower chronic pain patients for better self-management and more adequate coping strategies when exposed to pain. As HL is a competence that develops continuously, it is important to support this development during the therapy. This, of course, is a challenge for the attending physicians. The provided information should be prepared and communicated individually, according to personal needs and knowledge. Comprehension of the provided information should be ensured through the teach-back method and not be taken as a self-evident fact. Furthermore, patients searching for information should be actively supported by their physicians. Future research should investigate the personal attitude and competences of physicians about knowledge transfer with an individual approach and also including community-based initiatives and mass media campaigns and HL [43, 44]. Another aspect that should receive attention is the difference between the pain intensity measured by different instruments. Furthermore, the role and potential of self-help groups to help relieve the burden of chronic pain in Austria should be investigated.
